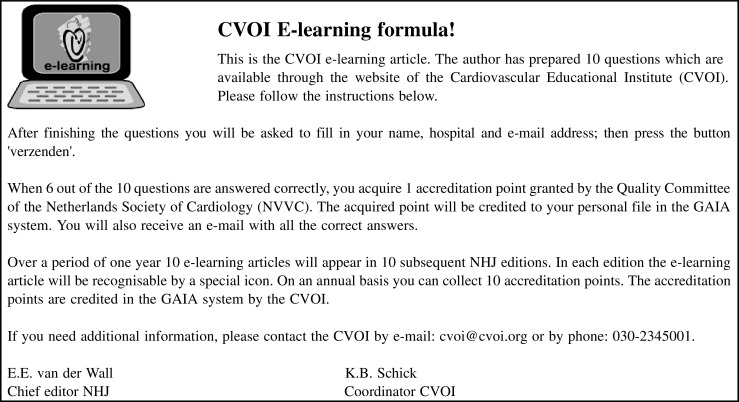# Direct haemodynamic effects of pulmonary arteriovenous malformation embolisation

**DOI:** 10.1007/s12471-014-0539-7

**Published:** 2014-03-07

**Authors:** V. M. M. Vorselaars, S. Velthuis, J. J. Mager, R. J. Snijder, W.-J. Bos, J. A. Vos, M. J. L. van Strijen, M. C. Post

**Affiliations:** 1 Department of Cardiology, St. Antonius Hospital, Koekoekslaan 1, 3435 CM Nieuwegein, the Netherlands; 2 Department of Pulmonology, St. Antonius Hospital, Nieuwegein, the Netherlands; 3 Department of Internal Medicine, St. Antonius Hospital, Nieuwegein, the Netherlands; 4 Department of Radiology, St. Antonius Hospital, Nieuwegein, the Netherlands

**Keywords:** Pulmonary arteriovenous malformation, Rendu-Osler-Weber syndrome, Hereditary haemorrhagic telangiectasia, Transcatheter embolisation, Haemodynamics, Finometer

## Abstract

**Background:**

Transcatheter embolisation is widely used to close pulmonary arteriovenous malformations (PAVMs) in patients with hereditary haemorrhagic telangiectasia (HHT). Data on the direct cardiovascular haemodynamic changes induced by this treatment are scarce.

**Objectives:**

We investigated the direct haemodynamic effects of transcatheter embolisation of PAVMs, using non-invasive finger pressure measurements.

**Methods:**

During the procedure, blood pressure, heart rate (HR), stroke volume (SV), cardiac output (CO), total peripheral resistance (TPR) and delta pressure/delta time (dP/dt) were continuously monitored using a Finometer®. Potential changes in these haemodynamic parameters were calculated from the pressure registrations using Modelflow® methodology. Absolute and relative changes were calculated and compared using the paired sample *t*-test.

**Results:**

The present study includes 29 HHT patients (mean age 39 ± 15 years, 11 men) who underwent transcatheter embolotherapy of PAVMs. The total number of embolisations was 72 (mean per patient 2.5). Directly after PAVM closure, SV and CO decreased significantly by −11.9 % (*p* = 0.01) and −9.5 % (*p* = 0.01) respectively, without a significant change in HR (1.8 %). Mean arterial blood pressure increased by 4.1 % (*p* = 0.02), while the TPR and dP/dt did not increase significantly (5.8 % and 0.2 %, respectively).

**Conclusions:**

Significant haemodynamic changes occur directly after transcatheter embolisation of PAVMs, amongst which a decrease in stroke volume and cardiac output are most important.

## Introduction

Hereditary haemorrhagic telangiectasia (HHT) is an autosomal dominant inherited disease characterised by vascular malformations, ranging from small telangiectases in skin and mucosal membranes to large visceral arteriovenous malformations (AVMs) predominantly localised in the lungs, brain and liver [1–3]. Pulmonary arteriovenous malformations (PAVMs) are abnormally dilated vessels between pulmonary arteries and veins that cause a permanent extra-cardiac right-to-left shunt, which carries the risk of cerebral paradoxical embolisation of both thrombotic and septic origin [2]. Transcatheter embolisation of PAVMs can be safely performed, in order to prevent these potentially severe neurological complications, such as ischaemic stroke or cerebral abscess [3, 4]. Currently, there are no data regarding the potential haemodynamic changes occurring directly after PAVM embolisation. Therefore, the present study investigated the direct haemodynamic effects of PAVM embolisation, using non-invasive finger pressure measurements.

## Methods

### Patient population

Between 2008 and 2010, we included 29 patients who underwent transcatheter embolisation of PAVMs in the St. Antonius Hospital Nieuwegein, which is a national HHT referral centre in the Netherlands. All patients provided informed consent.

### Transcatheter embolotherapy of PAVMs

**Fig. 1:**
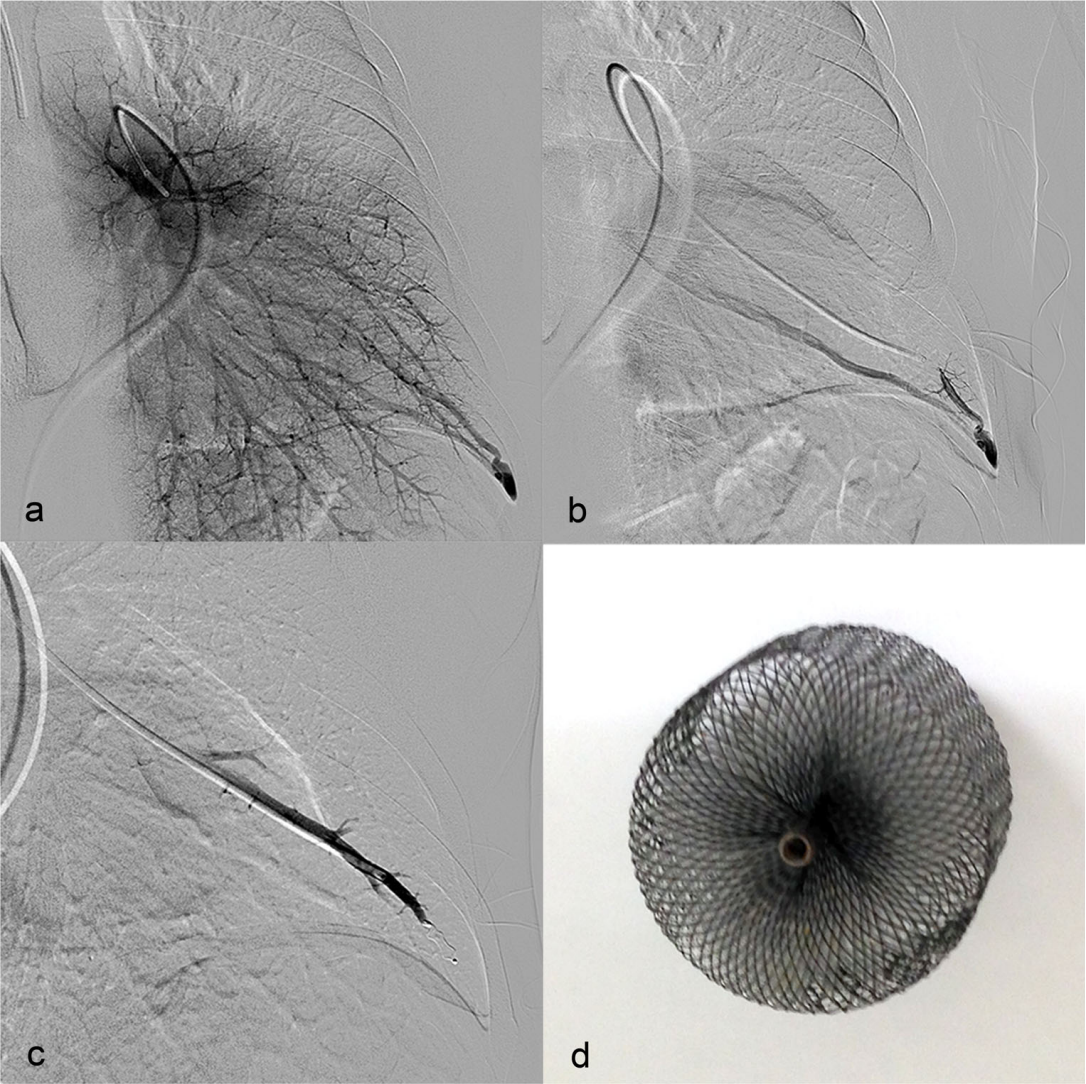
**a** Pulmonary angiogram of pulmonary arteriovenous malformation in the left lower lobe. **b** Selective angiogram of pulmonary arteriovenous malformation in the left lower lobe. **c** Embolisation of pulmonary arteriovenous malformation in the left lower lobe with a Amplatzer® vascular plug. **d** Amplatzer® vascular plug

A PAVM was defined as a direct communication between a pulmonary artery and a pulmonary vein, bypassing the pulmonary capillary filter [5], and was diagnosed using transthoracic contrast echocardiography (TTCE) and subsequent chest computed tomography (CT). Before and after PAVM embolisation, the right-to-left shunt fraction was measured using the 100 % oxygen method as previously described [6]. All patients were discussed in a multidisciplinary team including a pulmonologist and interventional radiologist. PAVMs with a feeding artery diameter of 2–3 mm or greater were found suitable for embolisation therapy [7]. The procedure (Fig. 1a–c) was performed under local anaesthesia (lidocaine 1 %). Percutaneous access was derived through the right femoral vein and a six French sheath was inserted. The interventional radiologist selected the PAVM closure device, based on the diameter and anatomy of the PAVM. The most preferred closure device was the Amplatzer® vascular plug (AGA Medical, Golden Valley, MN, USA) (Fig. 1d). Plugs with a diameter of 4–12 mm were used. If PAVM closure with a plug was not possible, detachable coils (Boston Scientific, Natick, Ma) were used. The embolic material was implanted under fluoroscopic guidance, with a maximum contrast volume of 300 ml (Xenetix; Iobitrol, Guerbet, Villepinte, France). Within 24 h after embolisation, a chest X-ray was performed. No standard medication was given.

### Haemodynamic changes after PAVM embolisation, using non-invasive finger pressure measurements

During the transcatheter embolisation of PAVMs, arterial pressure was measured on a finger of the left hand using a Finometer® device (FMS, Finapres Medical Systems, Amsterdam, the Netherlands). The Finometer® measures blood pressure by a combination of the volume clamp method of Penaz and the ‘Physiocal’ criteria developed by Wesseling [8–10]. The hand was kept at heart level and a cuff was wrapped around the same arm for individual blood pressure calibration using the return-to-flow calibration [8, 11]. Because of potential distortion of the measurements at the time of PAVM closure, parameters were recorded after stabilisation of the finger pressure signal during a blanking period of 1 min immediately before and after placement of the first and the last plug.

### Data registration and analysis

During the PAVM embolisation, finger pressure measurements with the associated event marks were monitored and digitally stored. Review of these data was performed using BeatScope® software. Five minute averages of systolic (SBP), diastolic (DBP) and mean blood pressure (MBP), heart rate (HR), stroke volume (SV), cardiac output (CO), total peripheral resistance (TPR) and delta pressure/delta time (dP/dt) were calculated after an electronic calibration procedure. SV and CO (CO is the product of SV and HR) were calculated from the finger pressure wave using the Modelflow® methodology [12]. The cardiac index (CI) was calculated from the CO and the body surface area. TPR was defined as MBP divided by CO [8].

Return-to-flow calibration, using the arm cuff, was used for calibration of the blood pressure. There was no calibration with invasive determinations for the CO, SV and TPR. Both absolute values and absolute and relative changes (delta absolute and delta percent) are presented.

### Statistical analysis

The statistics were performed using SPSS version 17.0 for Windows (SPSS Inc., Chicago, IL, USA). Descriptive statistics were used to describe patient characteristics. Continuous variables with normal distribution were presented as mean ± SD. Differences within groups were analysed performing paired samples *t*-tests. A significance level of *p* < 0.05 was considered significant.

## Results

### Patient population

A total of 29 HHT patients (62 % female, mean age 39.2 ± 15.3 years) were included, in which 72 PAVMs were embolised (mean per patient 2.5). An Amplatzer® plug was used in 54 cases and a detachable coil in the remaining 18 cases. The baseline characteristics are presented in Tables 1 and 2.

**Table 1:** Baseline characteristics of patients

Total		29
Gender	Male	11 (37.9)
	Female	18 (62.1)
Age (years)		39.2 ± 15.3
BMI (kg/m^2^)		24.0 ± 5.0
BSA (m^2^)		1.9 ± 0.2
HHT	Definite	28 (96.6)
	Type 1	19 (65.5)
	Type 2	2 (6.9)
	Type unknown	8 (27.6)
SaO2 (%)	Before procedure	95.5 ± 3.5
	After procedure	98.4 ± 2.3
Shunt fraction (%)	Before procedure	13.6 ± 8.3
	After procedure	4.9 ± 6.6

All characteristics are written in number of patients with percentage or mean with standard deviation

*BMI* body mass index, *BSA* body surface area, *Kg* kilogram, *kg/m* ^*2*^ kilogram per square meter, *HHT* hereditary haemorrhagic telangiectasia, *SaO*_*2*_ saturation level of oxygen in haemoglobin, *P AVM* pulmonary arteriovenous malformation

**Table 2:** Baseline characteristics of embolisation procedure

Treated PAVMs	1 PAVM	9 (31.0)
	2 PAVMs	9 (31.0)
	3 PAVMs	2 (6.9)
	4 PAVMs	8 (27.6)
	>4 PAVMs	1 (3.4)
Closure device	Amplatzer plug	54 (75.0)
	Coil	18 (25.0)
Plug diameter^a^	<4 (mm)	1 (1.9)
	4 (mm)	13 (24.1)
	6 (mm)	13 (24.1)
	8 (mm)	9 (16.7)
	10 (mm)	10 (18.5)
	>10 (mm)	6 (11.1)
	Not known	2 (3.7)

All characteristics are written in number with percentage

*PAVM* pulmonary arteriovenous malformation, *mm* millimetre

^a^Diameter coils are not know

### Haemodynamic changes using non-invasive finger pressure measurements

Directly after PAVM embolisation the SV and CO decreased significantly: −6.4 ± 13.0 ml (range −45.9 to 17.9 ml; −11.9 %, *p* = 0.01) and −0.4 ± 0.8 l/min (range −3.0 to 1.58 l/min; −9.5 %, *p* = 0.01). As expected, the CI decreased as well (range −1.5 to 0.9 l/min/m^2^; −9.5 %, *p* = 0.01). DBP and MBP increased significantly by 5.2 ± 10.3 mmHg (range −9.0 to 32.9 mmHg; 5.9 %, *p* = 0.01) for DBP and 5.7 ± 12.1 mmHg (range −12.7 to 32.6 mmHg; 4.1 %, *p* = 0.02) for MBP, respectively. There was no significant change in SBP (4.0 ± 16.3 mmHg (range −25.5 to 38.5 mmHg; 1.7 %, *p* = 0.20). The dP/dt did not change significantly: 2.1 ± 290.0 mmHg/s (range −750.0 to 848.2 mmHg/s; 0.2 %, *p* = 0.97). There was a correlation between the delta dP/dt and the SBP (Pearson coefficient *r* = 0.73, r^2^ = 0.53, *p* < 0.0001). HR and TPR increased, but this appeared to be non-significant: 1.6 ± 7.4 beats/min (range −17.4 to 21.2 beats/min; 1.8 %, *p* = 0.24) and 0.1 ± 0.5 Woods units (range −1.62 to 1.39 Woods units; 5.8 %, *p* = 0.16). These data are summarised in Table 3.

**Table 3:** Haemodynamic measurements before and after embolisation with absolute and relative changes

	Before ± SD	After ± SD	Delta absolute	Delta percent	P
SBP (mmHg)	144.9 ± 25.3	148.9 ± 28.4	4.0	1.7	0.20
DPB (mmHg)	85.5 ± 11.5	90.7 ± 16.3	5.2	5.9	0.01
MBP (mmHg)	108.0 ± 16.0	113.7 ± 19.9	5.7	4.1	0.02
HR (beats/min)	78.8 ± 14.6	80.4 ± 13.9	1.6	1.8	0.24
SV (ml)	70.9 ± 20.9	64.5 ± 19.3	−6.4	−11.9	0.01
CO (l/min)	5.5 ± 1.6	5.1 ± 1.4	−0.4	−9.5	0.01
CI (l/min/m^2^)	3.0 ± 0.8	2.8 ± 0.8	−0.2	−9.5	0.01
TPR (woods units)	1.4 ± 0.5	1.5 ± 0.5	0.1	5.8	0.16
dP/dt (mmHg/s)	1,233.7 ± 463.8	1,235.8 ± 481.7	2.1	0.2	0.97

*SBP* systolic blood pressure, *DBP* diastolic blood pressure, *MBP* mean blood pressure, *HR* heart rate, *SV* stroke volume, *CO* cardiac output, *CI* cardiac index, *TPR* total peripheral resistance, *dP/dt* delta pressure/delta time, *SD* standard deviation, *min* minutes, *ml* millilitres, *l/min* litres per minute, *mmHg* millimetres of mercury, *s* second

## Discussion

To our knowledge, this is the first study describing the occurrence of significant haemodynamic changes directly after transcatheter embolisation of PAVMs. Using the Finometer® and Modelflow® methodology, our study accurately recorded beat-to-beat non-invasive finger pressure measurements and thereby the immediate haemodynamic changes after PAVM embolisation, amongst which a decrease in SV and CO were most important.

Only one case report previously documented haemodynamic changes 4 months after PAVM embolisation with a marked reduction in CO of −5.1 l/min (41 %) [13]. This seems to be in line with the results in our current study in 29 HHT patients who all underwent transcatheter embolisation of PAVMs. However, we found a less pronounced decrease in CO, which can be explained by the smaller right-to-left shunts in our study population, with a mean shunt fraction before closure of 14 % versus 31 % described by Andrivet et al. [13]. It is possible that the CO may further decrease over time as a result of additional thrombosis of the plug or coil in the PAVM feeding artery. However, the long-term haemodynamic changes after PAVM embolisation remain hard to predict, since new PAVMs may occur and existing PAVMs may grow, so this is still subject for larger studies in the future.

The haemodynamic responses after PAVM embolisation may differ between HHT patients, which can be related to difference in number and size of PAVMs at baseline [2]. In the present study, nine patients underwent embolisation of at least 4 PAVMs in one session, whereas less PAVMs were embolised in the remaining 20 patients. Furthermore, a total of 16 PAVMs were treated with a large plug (diameter ≥10 mm), whereas 14 PAVMs could be treated with smaller endovascular plugs (diameter ≤4 mm). Unfortunately, we could not find a significant association between plug size (size of the PAVM) and the changes in haemodynamic parameters. Furthermore, the haemodynamic response can be influenced by a different prevalence of underlying hepatic arteriovenous malformations (HAVMs) in different HHT subtypes [5, 14, 15]. HAVMs may cause a hyperdynamic circulation with high CO [7]. In our cohort, only one patient had a history of HAVMs, which might be an underestimation, since screening for HAVMs was only performed when clinically or biochemically suspected. Clinically significant HAVMs seemed to be absent in the present study, as a hyperdynamic circulation with an abnormal high CI at baseline was not documented in the present study (mean CI within the normal range of 3.0 ± 0.8 l/min/m^2^). The amount of microscopic PAVMs under the detection limit of chest CT may further influence the shunt percentage and different haemodynamic responses in HHT patients.

Due to the decrease in pulmonary right-to-left shunt from 14 to 5 % after PAVM embolisation, a consequent decrease in preload and SV can be expected (mean change −12 % in the current study). As there was no change in HR, the CO decreased as well (−9.5 %). The MBP increased by 4 % after PAVM embolisation, which is probably due to the non-significant increase in TPR (6 %), as the MBP is a product of TPR and CO. As blood pressure is inversely related to indoor temperature [16], the fall in ambient temperature during the procedure may have caused further vasoconstriction and thereby an increase in blood pressure. In a previous study about the association between brachial pulse dP/dt and other haemodynamic parameters in a chronic haemodialysis population, a Pearson coefficient of *r* = 0.6 (r^2^ = 0.36, *p* < 0.001) was reported for the correlation between the delta blood pressure and the delta dP/dt [17]. This is in line with the results found in our study (*r* = 0.73, r^2^ = 0.53, *p* < 0.0001) and demonstrates that the dP/dt is responsible for more than 50 % of variance in the blood pressure. Because of the significant increase in MBP and decrease in CO, we also expected a significant increase in TPR. A possible explanation might be an increase in central venous pressure due to the embolisation. Unfortunately, no measurements of the right atrium pressure were performed during the embolisation procedure.

A potential clinical implication of our findings might be associated with the presence of pulmonary hypertension (PH) in HHT [18, 19]. PH can occur both as gene-related pulmonary arterial hypertension and as a response to high output due to HAVMs [14]. As PAVMs are abnormally dilated vessels between pulmonary arteries and veins they provide low resistance pathways for the pulmonary blood flow and one may therefore expect an elevation in pulmonary artery pressure (PAP) after transcatheter closure. Surprisingly, in a prior study by Shovlin et al. there was no described increase in PAP after transcatheter embolisation of PAVMs [14]. It was suggested that this might be caused by other haemodynamic changes, for example recruitment of the pulmonary vasculature or a decrease in CO, although this has been previously suggested in only one case report [13]. We now present the first study that confirms the decrease in CO after embolisation in a larger population of HHT patients, which may indeed provide a potential explanation for the absent increase in PAP after embolisation. Furthermore, PAVM-related hypoxaemia can induce pulmonary vasoconstriction with a concomitant increase in pulmonary vascular resistance (PVR). In our study there was indeed an increase in saturation after embolisation of PAVMs (Table 1) with probably an decrease in pulmonary vasoconstriction and PVR.

### Study limitations

First, our study is limited by its small sample, which may have influenced the results. Second, the Modelflow® model does not seem to be accurate in measuring absolute values of SV, CO and TPR. The differences between the uncalibrated model and invasive determinations (measured with the thermodilution method) in individual patients are usually small, but can be substantial and unreliable in some [8, 20]. However, the Modelflow® methodology is an accurate model to compare changes in haemodynamics within one patient [8] and the British Hypertension Society has recommended the Finometer® for measurements in the clinical set-up as well as for research purposes [8, 21].

## Conclusion

The present study shows that significant haemodynamic changes occur directly after embolisation of pulmonary arteriovenous malformations, amongst which a decrease in stroke volume and cardiac output are most important. This may especially provide additional insights into the haemodynamic responses after PAVM embolisation in HHT patients prone to PH.